# Simulation basis for a techno-economic evaluation of chitin nanomaterials production process using Aspen Plus® software

**DOI:** 10.1016/j.dib.2018.08.130

**Published:** 2018-08-31

**Authors:** Fatma Larbi, Araceli García, Luis J. del Valle, Ahmed Hamou, Jordi Puiggalí, Naceur Belgacem, Julien Bras

**Affiliations:** a Univ. Grenoble Alpes, CNRS, LGP2, F-38000 Grenoble, France; b IUF, F-75000 Paris, France; c Laboratory for study of Environmental Sciences and Materials (LESEM), El M’naouar, Oran, Algeria; d University of Oran 1 Ahmed Ben Bella, Department of Physics, El M’naouar, Oran, Algeria; e University of Cordoba, Department of Organic Chemistry, Marie Curie Building C-3, Crta Nnal IV km 396, 14014 Cordoba, Spain; f Barcelona Research Center for Multiscale Science and Engineering, Departament d׳Enginyeria Química, Escola d׳Enginyeria de Barcelona Est (EEBE), Univ. Politècnica de Catalunya (UPC), c/Eduard Maristany 10-14, 08019 Barcelona, Spain

## Abstract

Process simulation is a useful tool that has been widely used to analyze, design and optimize energy balances in chemical technologies including those related to biomass processing, biorefinery processes and chemical engineering. The presented data set serves as basis for the simulation of chitin purification, nanofibers and nanocrystals production processes, considering laboratory experimental procedures described in previous experimental articles.

**Specifications table**

**Table:** 

Subject area	*Chemistry*
More specific subject area	*Process Simulation, Chemical Engineering*
Type of data	*Figures, tables and text*
How data was acquired	*Process simulation software tool Aspen Plus (AspenTech, Virginia)*
Data format	*Simulation basis description*
Experimental factors	*For chitin thermochemical and physical properties, the estimation was carried out assuming them identical to those of cellulose (existing in Aspen database)*
Experimental features	*Common process modules and tools available in Aspen Plus were employed*
Data source location	*N/A*
Data accessibility	*N/A*

**Value of the data**

• There are no published data from a process engineering and economic standpoint on the cost production of such nanomaterials;
• Taking into account basic issues as water, chemicals and heating energy consumptions of the proposed processes;
• The here presented energy and economic evaluation of nanochitin production would help to estimate large-scale production costs;

## 1 Data

This article contains data related to the research article entitled “Comparison of nanocrystals and nanofibers produced from shrimp shell α-chitin: From energy production to material cytotoxicity and Pickering emulsion properties” [1]. The experimental methodology described herein served as basis for the simulation of chitin purification, chitin nanofibers (ChNFs) and chitin nanocrystrals (ChNCs) production processes for the present data article.

## 2 Experimental design, materials and methods

The following paragraphs briefly describe the methodology used for the development of a series of process simulations by using Aspen Plus® software.

As general considerations for simulation basis, 1 kg of chitin input was considered. Moreover, all the input streams (chemicals, water …) to the process were assumed at a temperature of 25 °C and 1 atm of pressure.

### 2.1 Simulation basis for Chitin Purification process

**Fig. 1:**
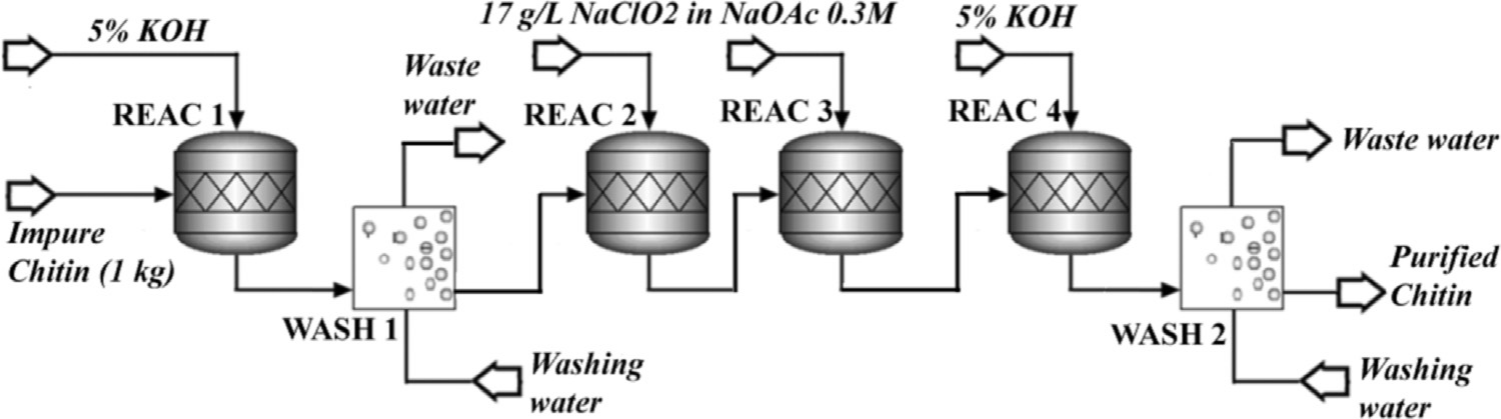
Flow sheet of the chitin purification step.

In Fig. 1 the flowsheet of the proposed chitin purification methodology is proposed. The process starts with an alkali pre-treatment (REAC 1: RStoic module), where the reactant is added in a liquid-to-solid mass ratio of 22.5 (5% w/w KOH). The reaction run at 100 °C and the 15% w/w of the chitin is supposed to dissolve. After that, the mixture is conducted to the washing/filtration step (WASH1: Swash module), operating at a liquid-to-solid mass ratio of 8 and with a mixing efficiency of 0.9. Water requirements in this module are determined by using a Design Spec tool, to reach a 2.0 ± 0.5% w/w of dissolved solids in the output stream.

Next step entails the bleaching of chitin (REAC 2: RStoic module), where the reactants are added in a liquid-to-solid mass ratio of 22.5 (17 g/L NaClO2 in NaOAc 0.3 M) and 2.5% w/w of the material is dissolved at 80 °C. The bleaching step is repeated (REAC 3: RStoic module) at the same conditions. The alkali pre-treatment is then repeated (REAC 4: RStoic module) with the same chemical load (liquid-to-solid mass ratio of 22.5 using 5% w/w KOH) but occurring at room temperature (25 °C) and with the 2.5% w/w of the material dissolved. Finally, a new washing/filtration step is carried out (WASH 2: Swash module) with the same specifications than WASH 1 module.

### 2.2 Simulation basis for ChNF preparation process

**Fig. 2:**
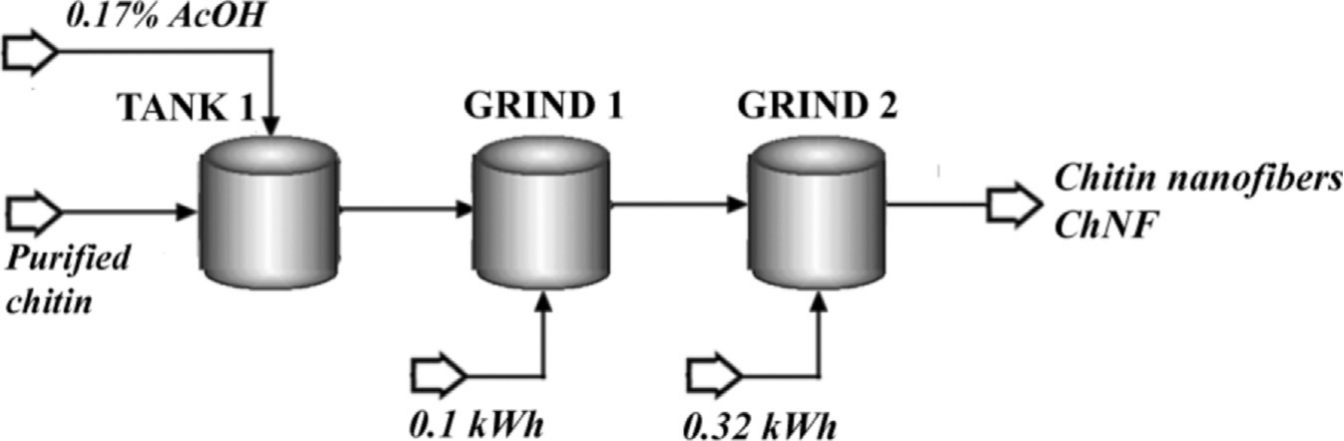
Flow sheet of the ChNF preparation process.

The proposed chitin nanofibers (ChNF) preparation process is shown in Fig. 2. As pre-treatment of the purified fibers resulted from the previous Chitin Purification step (Section 2.1.) a dilution and acidification of the fibers was conducted (TANK 1: Mixer module). The amount of acidified water (0.17% w/w of acetic acid AcOH) was calculated by using a Design Spec tool, to reach a 1.0 ± 0.1% w/w of fibers in the resulting suspension. After that, the grinding process was carried out (GRIND 1: Mixer module). This step is required during the experimental procedure for dispersion purposes, takes 10 min and acts as soft mechanical treatment. The simulation module contains in this case an energy input for calculation, and was estimated as 0.1 kWh from [2]. A second mechanical treatment was specified in GRIND 2 (Mixer module) in order to simulate the ultrafine grinding or high performance/speed mechanical operation for defibrillation. This step required 3 h, with an energy consumption of 0.318 kWh/kg of purified chitin, experimentally determined [1]

### 2.3 Simulation basis for ChNC preparation process

**Fig. 3:**
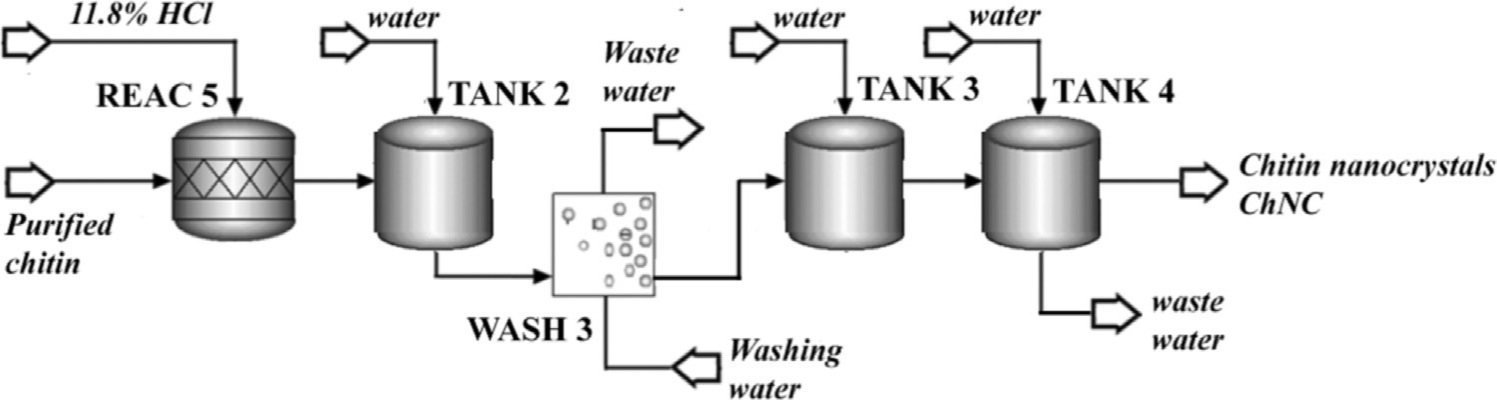
Flow sheet of the ChNF preparation process.

According to the methodology described in [1], the chitin nanocrystals production process began with a three-step chemical treatment of the previously purified chitin fibers. As detailed in Fig. 3, the acidic hydrolysis of chitin was performed using an acid-to-chitin mass ratio of 30 (11.8% HCl), in a reactor (REAC 5: RStoic module) at 90 °C during 90 min, where the 80% of the purified chitin is transformed into nanocrystals (according to experimental data). After that, the stream was diluted with 2 volumes of water (TANK 2: Mixer module) for reaction quenching. The resulted treated chitin was then 3 times washed/filtrated (WASH 3: Swash module) using water at a liquid-to-solid mass ratio of 8 and mixing efficiency of 0.9. Water requirement was determined by using a Design Spec tool, to reach a 1.0 ± 0.5% of dissolved solids in the output stream. This procedure was conducted by triplicate.

After the above described treatment, the nanocrystals containing stream was diluted in TANK 3 (Mixer module), where water requirement was determined by using Design Spec tool, to reach a 1.5 ± 0.5% of solids in the output stream. Finally, the dialysis of the ChNC suspension was performed in TANK 4 (SSplit module) using 20 times volume of water. The efficiency of the purification was established according to the dilution.

### 2.4 Economic considerations

In order to evaluate economic aspects of the proposed nanochitin production process, energy [3], water [4] and chemicals expenses [5] were considered and taken into account together with simulation resulted energy and mass balances. These costs are detailed in Table 1.

**Table 1:** Cost related to energy, water and chemicals considered for the techno-economic evaluation of nanochitin production process.

	**Energy (kW)**	**Water (m**^**3**^**)**	**Chemicals (kg)**
***Cost per unit (€/unit)***	*0.058*	*0.850*	*1.346 (AcOH)*
			*0.871 (KOH 92%)*
			*0.078 (HCl 22°Be)*
			*3.088 (NaClO*_*2*_*80%)*
			*0.783 (NaOAc)*
